# Impact of an Individualized Dietary Intervention on Body Composition and Clinical Outcomes in Patients with Neovascular Age-Related Macular Degeneration: A Pilot Study

**DOI:** 10.3390/nu18121870

**Published:** 2026-06-10

**Authors:** Daria Szulim, Elżbieta Kucharska, Anna Machalińska, Leszek Kuprjanowicz, Piotr Czupryński, Małgorzata Szczuko

**Affiliations:** 1 Department of Bromatology and Nutritional Diagnostics, Pomeranian Medical University in Szczecin, 70-111 Szczecin, Poland; 2 Clinical Trials Support Center at the Pomeranian Medical University in Szczecin, ul. Unii Lubelskiej 1, 71-252 Szczecin, Poland; piotr.czuprynski@pum.edu.pl; 3 Department of Commodity Science, Quality Assessment, Process Engineering and Human Nutrition, West Pomeranian University of Technology in Szczecin, Kazimierza Królewicza St. 4, 71-550 Szczecin, Poland; elzbieta.kucharska@zut.edu.pl; 4 First Department of Ophthalmology, Pomeranian Medical University, Al. Powstańców Wielkopolskich 72, 70-111 Szczecin, Poland; anna.machalinska@pum.edu.pl (A.M.); leszek.kuprjanowicz@pum.edu.pl (L.K.)

**Keywords:** age-related macular degeneration, AMD, diet, nutritional intervention, body composition, anthropometry, anti-VEGF therapy, carotenoids

## Abstract

**Background/Objectives**: Age-related macular degeneration (AMD) is a leading cause of permanent deterioration of central vision in elderly people. While anti-VEGF therapy remains the standard of care for neovascular AMD, disease progression and functional deterioration remain common. **Methods**: This pilot prospective controlled study evaluated the effects of an individualized dietary intervention in patients with neovascular AMD receiving anti-VEGF therapy. A total of 43 patients completed a six-month follow-up and were divided into a control group (standard treatment) and an intervention group receiving a personalized dietary plan. Anthropometric and body composition parameters were assessed using bioelectrical impedance analysis (BIA). Clinical retinal outcomes were classified as improvement, no change, or deterioration. Statistical analyses included parametric and non-parametric tests. **Results**: Retinal outcomes did not differ significantly between groups, although patients with retinal improvement or stabilization in the intervention group tended to exhibit more favorable changes in body composition. After adjustment for baseline values using analysis of covariance (ANCOVA), the dietary intervention remained significantly associated with reductions in body weight and BMI (*p* < 0.01). After 6 months, the intervention group demonstrated a numerically higher frequency of retinal improvement and a lower frequency of retinal deterioration compared with the control group; however, these differences were not statistically significant (*p* = 0.386). **Conclusions**: An individualized dietary intervention effectively improved body composition in patients with neovascular AMD but did not produce a statistically significant effect on retinal status over six months. Nevertheless, the dietary intervention group showed numerically more favorable retinal outcomes than the control group. Dietary modification may support general health and weight management.

## 1. Introduction

Age-related macular degeneration (AMD) is a chronic, progressive disease of the central retina and one of the leading causes of irreversible visual impairment among older adults in developed countries [1,2,3]. Despite advances in ophthalmological treatment, especially anti-VEGF therapy, disease control and preservation of central vision still pose a significant clinical challenge [4,5,6].

AMD is broadly classified into two major forms: dry (atrophic) and wet (neovascular) AMD. Dry AMD is the more common form and is characterized by gradual retinal pigment epithelium degeneration and drusen formation, whereas neovascular AMD is associated with choroidal neovascularization, retinal fluid accumulation, hemorrhage, and rapid vision loss [7].

Growing evidence suggests that modifiable lifestyle factors, including diet, nutritional status, and body composition, play a role in the development and progression of AMD. Epidemiological studies indicate that obesity and metabolic disturbances are associated with a higher risk of disease progression, potentially through mechanisms involving chronic inflammation, oxidative stress, and altered bioavailability of macular carotenoids such as lutein and zeaxanthin [8,9,10,11,12].

Lutein and zeaxanthin form the macular pigment in the retina, where they absorb blue light (400–500 nm) and protect against phototoxic retinal damage. Acting as optical filters, they improve contrast sensitivity and reduce glare, while also exerting antioxidant and anti-inflammatory effects [13,14]. Lutein is a xanthophyll carotenoid that accumulates in high concentrations in the macula of the human retina. As lutein cannot be synthesized de novo in the human body, it must be obtained from dietary sources, particularly green leafy vegetables and egg yolks. Lutein exhibits antioxidant and anti-inflammatory properties and plays an important role in protecting retinal tissues from oxidative stress and light-induced damage. Previous experimental and clinical studies suggest that lutein intake may have a protective role in age-related macular degeneration and other ocular diseases [15]. Carotenoids, including lutein and zeaxanthin, are lipophilic compounds that accumulate in adipose tissue. Increased adipose tissue may act as a reservoir for carotenoids, potentially affecting their bioavailability and distribution to retinal tissues. A potential mechanism underlying the relationship between diet and AMD progression may involve interactions between dietary intake, adipose tissue, and carotenoid bioavailability. Adipose tissue may serve as a reservoir for lipophilic carotenoids, such as lutein and zeaxanthin, thereby altering their systemic distribution and reducing their availability for retinal uptake. Consequently, this may enhance oxidative stress and inflammatory processes within the retina, potentially contributing to AMD progression [16].

Nutritional interventions aimed at slowing AMD progression have been investigated mainly through dietary supplementation. Large clinical trials, including the Age-Related Eye Disease Study (AREDS) and AREDS2, demonstrated that selected antioxidant supplementation may reduce the risk of progression to advanced AMD in specific patient groups [17,18]. However, increasing attention is being paid to overall dietary patterns, as diets rich in fruits, vegetables, whole grains, and fish have been associated with a lower risk of advanced AMD [19,20,21].

Despite these findings, data on individualized dietary interventions tailored to the specific nutritional needs of elderly patients remain limited. Older adults often present with food intolerances, comorbidities, reduced appetite, and selective eating habits due to difficulties in chewing or swallowing, which can lead to nutritional deficiencies and further complicate the design of effective dietary interventions [22,23].

The aim of this study was to implement an individualized diet rich in carotenoids and bioactive compounds with anti-inflammatory properties. A six-month observation was conducted to evaluate its effects on body composition and clinical outcomes in patients with neovascular AMD receiving anti-VEGF therapy.

## 2. Materials and Methods

### 2.1. Study Population

#### Inclusion and Exclusion Criteria

Initially, 89 patients aged over 65 years with clinically diagnosed age-related macular degeneration (AMD) were enrolled in the observational phase of the study.

All study procedures, including patient recruitment, clinical diagnosis, administration of intravitreal therapy, and follow-up assessments, were conducted at the First Department of Ophthalmology, Pomeranian Medical University, Szczecin, Poland.

Patients were eligible for inclusion if they were ≥65 years of age, had clinically confirmed neovascular (exudative) age-related macular degeneration (AMD), were treated with intravitreal bevacizumab (Avastin), qualified for therapy according to current clinical guidelines, and agreed to participate in the study for six months. Patients with neovascular AMD were included regardless of disease stage if they were receiving intravitreal anti-VEGF therapy. Ophthalmic outcomes were subsequently evaluated individually by an ophthalmologist based on clinical examination, OCT, and angio-OCT findings, with retinal status classified as improvement, stabilization/no progression, or deterioration after 6 months of follow-up.

The presence of diet-related chronic diseases, such as type 2 diabetes, arterial hypertension, or atherosclerosis, did not constitute an exclusion criterion.

Patients were excluded if they were younger than 65 years, had dry (non-exudative) AMD, had neovascular AMD not qualifying for intravitreal treatment, or were treated with anti-VEGF agents other than bevacizumab. Additional exclusion criteria included the use of dietary supplements that could influence the measured biochemical parameters, the presence of other ocular diseases affecting retinal function or visual acuity, severe systemic diseases potentially affecting nutritional status or body composition (e.g., active malignancies, advanced renal or hepatic failure, gastrointestinal diseases with malabsorption), and inability to complete the six-month follow-up.

**Figure 1:**
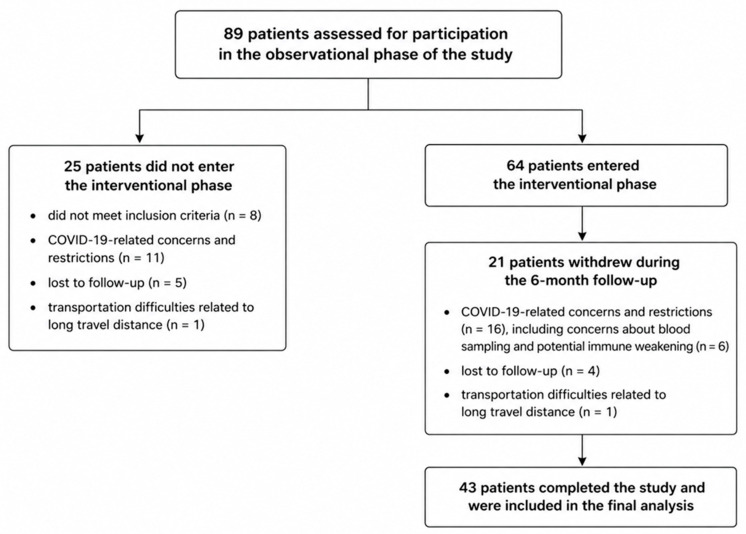
CONSORT-style flow diagram of participant recruitment, follow-up, withdrawals, and inclusion in the final analysis.

A total of 64 patients entered the interventional phase; however, only 43 patients completed the six-month follow-up and were included in the final analysis (Figure 1).

The study was conducted in a single clinical center and included consecutively recruited patients receiving anti-VEGF therapy, which may have influenced baseline differences between groups and could limit the broader generalizability of the findings.

Withdrawals were mainly associated with COVID-19-related concerns and restrictions during the pandemic period, including concerns related to blood sampling and potential immune weakening among some elderly participants. Many participants were reluctant to attend the scheduled six-month follow-up visits due to concerns about SARS-CoV-2 exposure. Additional reasons included loss to follow-up and transportation difficulties related to long travel distance. No participants reported withdrawal due to difficulties directly related to the dietary intervention itself.

An additional limitation of the study was the lack of inclusion of biochemical markers related to carotenoid status and oxidative stress in the present analysis. Although blood samples were collected for assessment of lutein, zeaxanthin, lycopene, antioxidant enzymes, and oxidative stress markers, these analyses are still ongoing and will be presented in a separate publication. Consequently, adherence to the dietary intervention was assessed through regular dietary monitoring and participant self-report during follow-up contacts and clinical visits, which may be subject to recall and reporting bias.

All participants received intravitreal bevacizumab injections (1.25 mg). Participants were divided into two groups: a control group receiving standard anti-VEGF therapy alone and an intervention group receiving standard anti-VEGF therapy combined with an individualized dietary plan.

### 2.2. Dietary Intervention

Patients in the interventional group followed an individualized, balanced dietary plan for six months. Diets were designed using dedicated Tiqdiet software (TiqDiet Polska Sp. z o.o., Szczecin, Poland) an online nutrition planning platform and tailored to age, sex, nutritional status, comorbidities, food intolerances, allergies, food aversions, and economic constraints. Meal plans were individualized regarding energy content (based on total energy expenditure) and macronutrient composition. The dietary intervention emphasized increased intake of nutrients potentially important for retinal health, including lutein, zeaxanthin, lycopene, vitamins A, C, and E, zinc, copper, manganese, selenium, and omega-3 fatty acids.

Participants received a 30-day rotating meal plan, dietary counseling, and detailed instructions regarding food preparation and portion sizes.

Patients in the intervention group were instructed to follow their individualized daily meal plans. They were provided with the study researcher’s contact information (phone and email) and could reach out at any time for questions or clarifications. Adherence to the dietary intervention was monitored through regular telephone contact with the study researcher, initiated either by the researcher or the patient, and tailored to participants’ individual needs and dietary support requirements.

Patients also attended monthly clinical visits at the ophthalmology clinic for intravitreal injections or routine ophthalmologic examinations. During these visits, the study researcher assessed dietary adherence, asking patients whether they followed the meal plans, whether the daily rations were manageable, and whether any adjustments were needed. Self-reported adherence was declared by the participants with the provided meal plans and reported consuming the full daily rations.

The diets were designed to meet daily nutrient requirements and included specific amounts of key micronutrients and bioactive compounds (Table 1). On average, the diets contained: lutein and zeaxanthin (6 mg), lycopene (5–10 mg), vitamin E (8 mg/day for women, 10 mg/day for men), zinc (8 mg/day for women, 11 mg/day for men), copper (0.9 mg/day), manganese (1.6–1.8 mg/day for women, 2.1–2.3 mg/day for men), vitamin C (75 mg/day for women, 90 mg/day for men), omega-3 fatty acids (EPA + DHA, 250–500 mg/day), selenium (55 µg/day), and vitamin A (700 µg/day for women, 900 µg/day for men) [24,25,26,27,28].

The control group received no dietary recommendations beyond standard clinical care.

**Figure 2:**
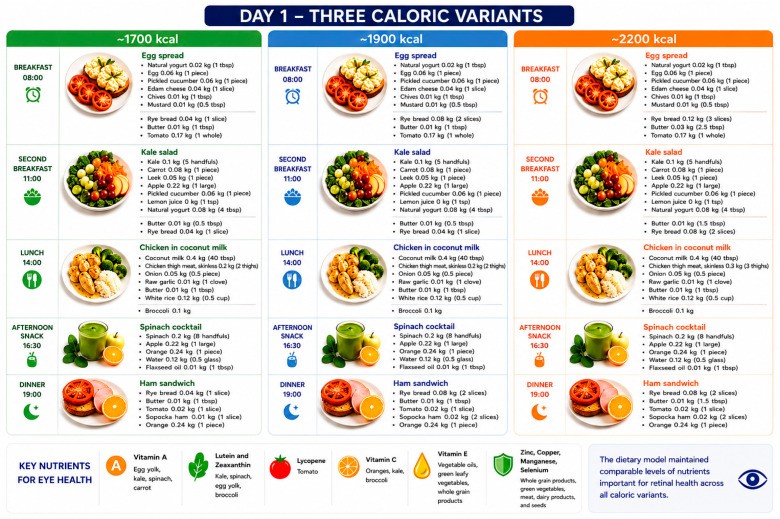
Representative one-day meal plans for three caloric variants (1700, 1900, and 2200 kcal) used in the individualized dietary intervention. The dietary model maintained comparable levels of nutrients important for retinal health across all caloric variants.

In the intervention group, dietary plans differed in total caloric value and macronutrient composition according to individual energy requirements. However, all diet variants were based on the same nutritional model and were designed to provide comparable levels of carotenoids, antioxidant vitamins (A, C, and E), zinc, copper, manganese, and selenium through the consistent inclusion of nutrient-rich foods (Figure 2).

Along with the individualized meal plans, participants received dietary recommendations and grocery lists, including detailed recipes, meal preparation instructions, recommended food products, and practical household portion guidance. The dietary intervention emphasized increased consumption of foods naturally rich in bioactive nutrients potentially involved in retinal protection and oxidative stress reduction in AMD, including lutein, zeaxanthin, lycopene, antioxidant vitamins, and trace elements. The 1900 kcal diet served as the reference model for nutrient composition analysis, while the remaining caloric variants were proportionally adjusted according to individual energy requirements within the same dietary framework.

The applied dietary model may also be considered a nutraceutical-oriented dietary approach, as it emphasized whole foods naturally rich in bioactive compounds with potential antioxidant and retinal-protective properties. Rather than focusing on isolated supplementation, the intervention relied on synergistic interactions between carotenoids, antioxidant vitamins, trace elements, fiber, and omega-3 fatty acids delivered through whole foods. Such dietary patterns share several characteristics with the Mediterranean diet, which has been associated with reduced oxidative stress, chronic inflammation, and age-related neurodegenerative processes. These nutrients may support retinal homeostasis by reducing oxidative stress and chronic inflammation, improving macular pigment stability, and supporting retinal metabolism. Therefore, dietary intervention may potentially contribute to stabilization of retinal condition and support anti-VEGF therapy in patients with neovascular AMD [29,30,31,32].

### 2.3. Anthropometric and Body Composition Measurements

Anthropometric measurements were performed at baseline and after six months. Body weight, body mass index (BMI), body fat percentage, visceral fat percentage, muscle mass, total body water, and metabolic age were assessed using bioelectrical impedance analysis (BIA) with a Tanita BC-545N analyzer (Tanita Corporation, Tokyo, Japan). Waist and hip circumferences were measured using an ergonomic anthropometric tape (seca 203; seca GmbH & Co. KG, Hamburg, Germany), and the waist-to-hip ratio (WHR) was calculated.

All participants were measured in the morning in a dedicated dietetic assessment room created for the study. Measurements were performed after participants had emptied their bladder, removed shoes and outer clothing, remaining in underwear only. Body height was measured using a Tanita HR-001 stadiometer (TANITA Polska, Szczecin, Poland).

Measurement accuracy: body weight: 0.1 kg; body fat and total body water: 0.1%; muscle mass: 0.1 kg (Tanita analyzer). Height and waist and hip circumferences (for WHR) were measured with 0.5 cm accuracy.

Body composition was assessed using bioelectrical impedance analysis (BIA), a non-invasive technique estimating fat mass, lean mass, and total body water by measuring electrical impedance. Participants were instructed to avoid heavy meals, alcohol, and intense exercise for at least 2–4 h prior to measurement.

Participants were screened for contraindications: none had implanted electronic devices or metal prostheses. All female participants were postmenopausal.

### 2.4. Clinical Ophthalmological Assessment

Ophthalmological evaluation including patient history, refraction (Topcon autorefractometer, Topcon Corporation, Tokyo, Japan) and visual acuity test (Snellen chart, ETDRS chart), intraocul pressure measurement (iCare tonometer, Icare Finland Oy, Vantaa, Finland), anterior segment examination using slit lamp and direct ophthalmoscopy (Volk 90D lens, Volk Optical Inc., Mentor, OH, USA) was performed by an ophthalmologist at baseline and after six months of follow-up. Optical coherence tomography (OCT) and OCT angiography (OCTA) images were obtained using the Spectralis OCT/OCTA system (Heidelberg Engineering, Heidelberg, Germany). Retinal assessment included evaluation of central retinal thickness (CRT measured in micrometers in central point of macula-“fovea centralis”), retinal morphology, intraretinal and subretinal fluid, pigment epithelial detachment, outer retinal layer integrity, and features of macular neovascularization. Disease activity and retinal status were assessed based on the overall interpretation of clinical examination and OCT/OCTA findings, including changes in central retinal thickness, retinal fluid, retinal morphology, and features of macular neovascularization. Ophthalmic outcomes were subsequently classified as improvement (1), stabilization/no progression (0), or deterioration (−1) of retinal status after six months of follow-up. All images were evaluated by the same ophthalmologist to ensure consistency of interpretation. Differences in categorical ophthalmological outcomes (improvement, stabilization/no progression, or deterioration of retinal status) between groups were assessed using Fisher’s exact test.

### 2.5. Blood Samples Collected

Blood samples were collected to determine concentrations of vitamins A and E, lutein, zeaxanthin, lycopene, zinc, copper, and manganese, as well as erythrocyte antioxidant enzyme activities (superoxide dismutase [SOD], catalase [CAT], glutathione peroxidase [GSH-Px]) and malondialdehyde (MDA) levels; these data are not included in the current analysis and will be evaluated later.

### 2.6. Statistical Analysis

Statistical analyses were performed using Statistica (version 13.1; TIBCO Software Inc., Palo Alto, CA, USA). Data distribution was assessed with the Shapiro–Wilk test, and homogeneity of variances was evaluated using Levene’s test. Depending on the data distribution, parametric tests (Student’s *t*-test) or non-parametric tests (Wilcoxon signed-rank test for paired samples and Mann–Whitney U test for independent samples) were applied.

Correlations between anthropometric parameters were assessed using Spearman’s rank correlation coefficients. A *p*-value < 0.05 was considered statistically significant.

To account for significant baseline differences in body weight and BMI between groups, an Analysis of Covariance (ANCOVA) was performed with post-intervention values as the dependent variable, group allocation (control vs. intervention) as the fixed factor, and corresponding baseline values as the covariate. Partial eta-squared (η^2^) was reported as a measure of effect size, with η^2^ ≥ 0.14 considered a large effect [33].

## 3. Results

A total of 43 patients with neovascular age-related macular degeneration completed the six-month study. Participants were divided into a control group (standard anti-VEGF therapy without dietary intervention) and an intervention group receiving an individualized dietary plan in addition to standard treatment. The mean age of participants was above 65 years in both groups, with a predominance of female patients. Baseline anthropometric parameters differed significantly between groups, with higher body weight and BMI observed in the intervention group (*p* < 0.05).

### 3.1. Changes in Body Weight

At baseline, body weight was significantly higher in the intervention group compared with the control group (81.02 ± 15.84 kg vs. 68.59 ± 8.74 kg, *p* < 0.05). After six months, the control group demonstrated a significant increase in body weight (from 68.59 ± 8.74 kg to 71.19 ± 9.59 kg, *p* < 0.05). In contrast, a significant reduction in body weight was observed in the intervention group (from 81.02 ± 15.84 kg to 77.93 ± 15.90 kg, *p* < 0.05) (Table 2).

**Figure 3:**
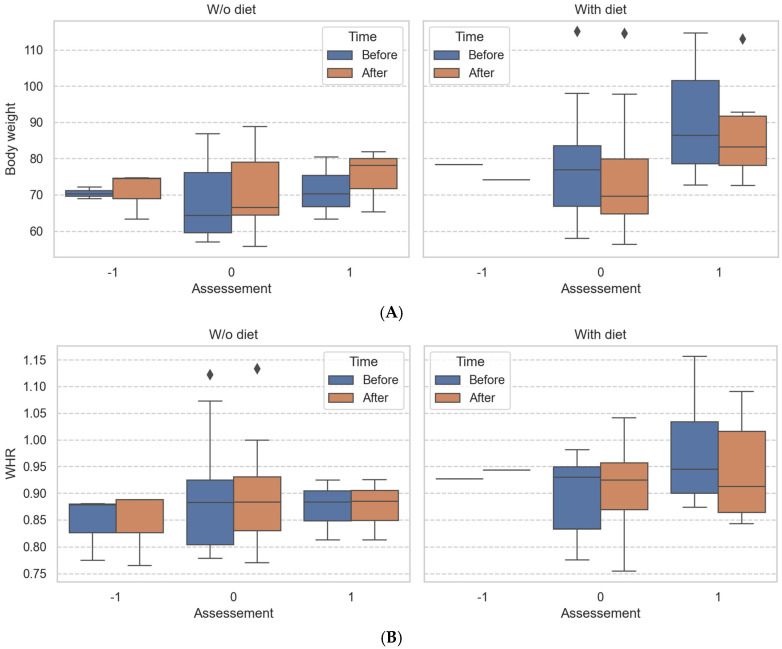

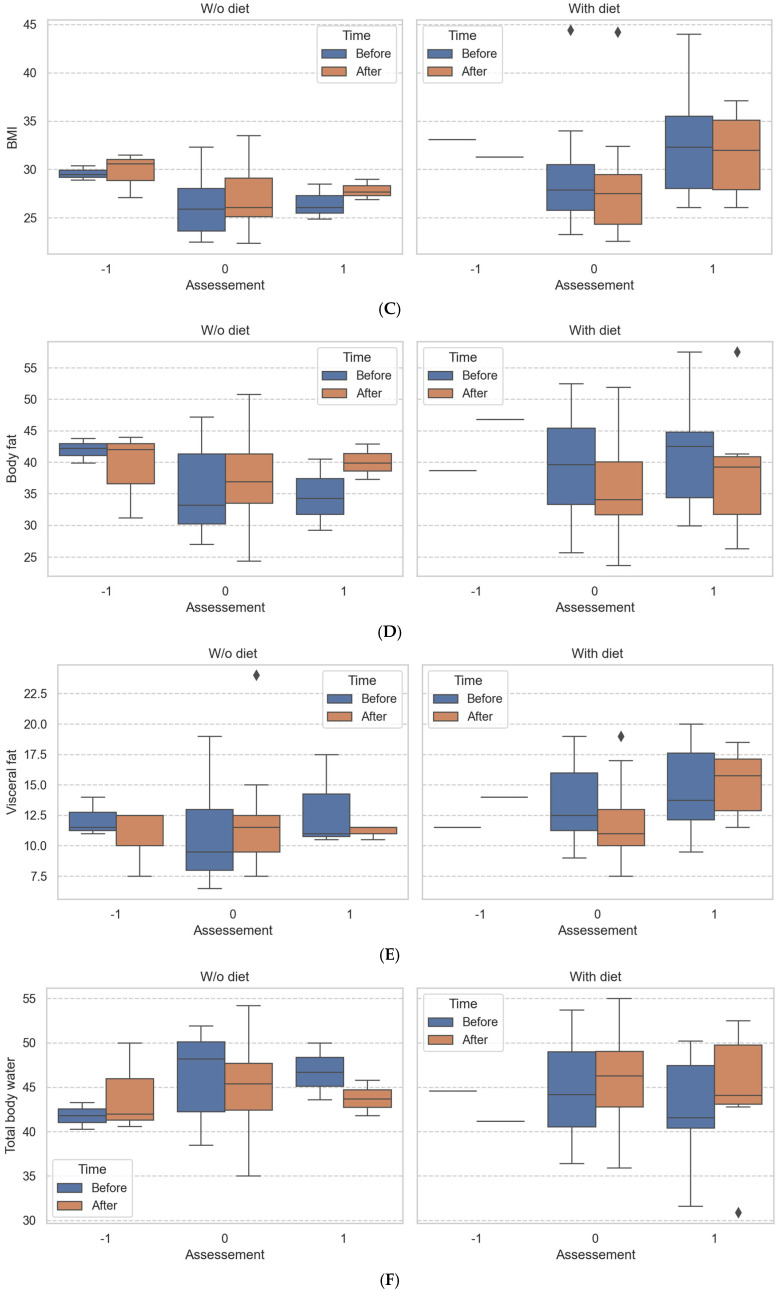

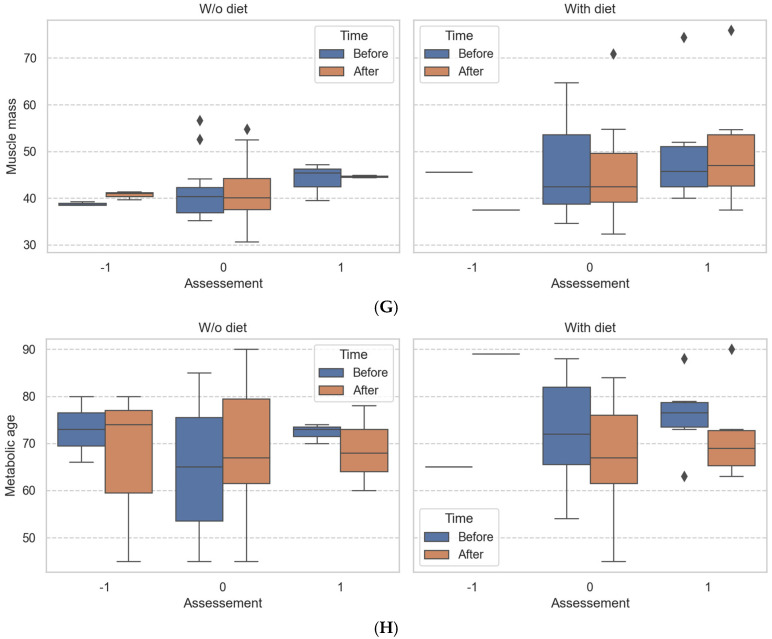
Changes in body weight, WHR, BMI, body fat, visceral fat, total body water, muscle mass and metabolic age in the control and study groups among patients with AMD with deterioration, no change, and improvement of retinal condition. (**A**) Body weight; (**B**) WHR; (**C**) BMI; (**D**) Body fat; (**E**) Visceral fat; (**F**) Total body water; (**G**) Muscle mass; (**H**) Metabolic age. −1—patients with deterioration of retinal condition; 0—patients with no changes in retinal condition; 1—patients with improvement of retinal condition.

When stratified according to clinical outcome (improvement, no change, or deterioration of retinal status), body weight increased across all outcome categories in the control group (Figure 3A). In the intervention group, a reduction in median body weight was observed in patients with improvement, no change, and deterioration of retinal status. Reference norm values for the Polish population, dependent on age and sex, were adopted: 45–75 kg for women and 55–85 kg for men [34].

### 3.2. Body Mass Index (BMI)

At baseline, BMI was significantly higher in the intervention group compared with the control group (30.42 ± 5.68 kg/m^2^ vs. 26.80 ± 2.83 kg/m^2^, *p* < 0.05). After six months, BMI increased slightly in the control group, whereas a significant decrease was observed in the intervention group (from 30.42 ± 5.68 kg/m^2^ to 29.17 ± 5.15 kg/m^2^, *p* < 0.05) (Table 2).

Analysis according to clinical outcome revealed a reduction in median BMI in the intervention group among patients with retinal improvement and deterioration, while no marked changes were observed in patients without clinical progression (Figure 3B). In interpreting the obtained BMI values of the patients, nutritional standards established for the Polish population were applied [34].

### 3.3. Waist-to-Hip Ratio (WHR)

At baseline, the waist-to-hip ratio (WHR) was significantly higher in the intervention group compared with the control group (0.92 ± 0.08 vs. 0.88 ± 0.09, *p* < 0.05). No statistically significant changes in WHR were observed in either group after six months of follow-up (Table 2).

Stratification by clinical outcome revealed minor variations in WHR, which were within normal ranges and did not show notable differences between subgroups (Figure 3C). The following WHR reference values were adopted for comparison with the obtained results: <0.8 for women and <1.0 for men [35]. Values exceeding these thresholds indicated an increased risk of health problems associated with abdominal obesity.

### 3.4. Body Fat and Visceral Fat

At baseline, no statistically significant differences in total body fat percentage were observed between the control and intervention groups (Table 2). After six months, no significant changes in total body fat percentage were detected in either group when analyzed as a whole.

Visceral fat percentage was significantly higher in the intervention group compared with the control group at baseline (13.70 ± 3.35% vs. 11.40 ± 3.42%, *p* < 0.05). After six months, no statistically significant changes in visceral fat percentage were observed in either group.

When stratified according to clinical outcome, distinct patterns were observed (Figure 3D). In the intervention group, a reduction in median body fat percentage was noted in patients with clinical improvement and in those without disease progression, whereas an increase was observed in patients with retinal deterioration. In contrast, the control group demonstrated an increase in median body fat percentage among patients with improvement and no change in retinal status, with no relevant changes in patients with deterioration. In analyzing the results for body fat percentage and visceral fat in patients, reference norms provided for the body composition analyzer Tanita BC-545N used in the study were applied [36].

### 3.5. Total Body Water

At baseline, total body water percentage was below reference values in more than half of the study population. No statistically significant differences were observed between the control and intervention groups at baseline or after six months of follow-up (Table 2).

Stratification by clinical outcome showed an increase in median total body water percentage in the intervention group among patients with retinal improvement and no change, whereas a decrease was observed in patients with retinal deterioration (Figure 3E). In the control group, a decrease in median total body water percentage was noted in patients with improvement and no change, while no relevant changes were observed in patients with deterioration. In analyzing the results for total body water in patients, reference norms provided for the body composition analyzer Tanita BC-545N used in the study were applied [36].

### 3.6. Muscle Mass

At baseline, muscle mass was significantly higher in the intervention group compared with the control group (46.67 ± 9.75 kg vs. 41.38 ± 5.43 kg, *p* < 0.05) (Table 2). After six months, no statistically significant changes in muscle mass were observed in either group.

Outcome-based analysis demonstrated an increase in median muscle mass in the intervention group among patients with retinal improvement (Figure 3F). In contrast, a decrease in median muscle mass was observed in patients with retinal deterioration in the intervention group. In the control group, minor changes in muscle mass were observed across outcome categories. The analysis of muscle mass results in patients was conducted based on the reference standards applicable to the Tanita BC-545N body composition analyzer used in the study [36].

### 3.7. Metabolic Age

No statistically significant differences in metabolic age were observed between the groups either at baseline or after six months of follow-up (Table 2).

Clinical outcome analysis demonstrated a decrease in the median metabolic age in both groups of patients who showed improvement in retinal status (Figure 3G). In contrast, divergent trends were observed among patients with no clinical changes and those with disease progression. In the intervention group, patients without clinical improvement exhibited a reduction in median metabolic age, whereas an increase was observed in the control group. In patients with retinal deterioration, an increase in metabolic age was noted in both groups. The interpretation of metabolic age results was based on the principles established by the manufacturer of the Tanita BC-545N body composition analyzer, according to which metabolic age is calculated by comparing an individual’s basal metabolic rate (BMR) with reference BMR values for specific age groups [36].

### 3.8. Adjusted Analysis Accounting for Baseline Differences

**Figure 4:**
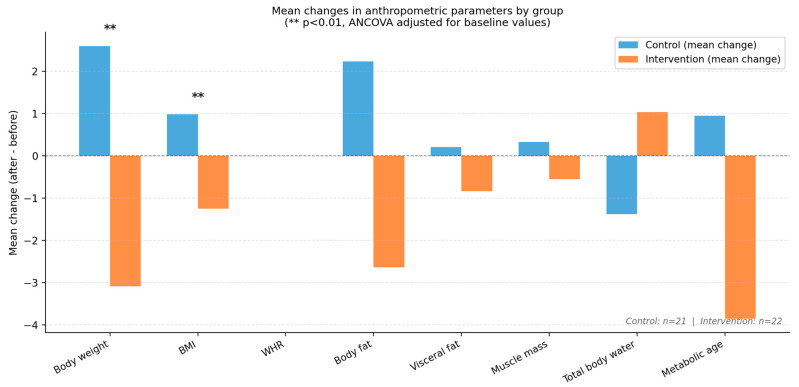
Mean changes in anthropometric and body composition parameters in the control and intervention groups after six months of follow-up. ANCOVA adjusted for baseline values was applied due to significant baseline differences between groups.

Given the significant baseline differences in body weight and BMI between groups, ANCOVA was performed to isolate the effect of dietary intervention from the influence of pre-existing anthropometric differences. After adjusting for baseline body weight, the intervention effect remained highly significant (F = 16.45, *p* = 0.0002, partial η^2^ = 0.29). The control group demonstrated a mean weight gain of +2.60 kg over six months, whereas the intervention group achieved a mean weight reduction of −3.09 kg (Figure 4). Similarly, the intervention effect on BMI remained significant after baseline adjustment (F = 12.60, *p* = 0.001, partial η^2^ = 0.24), with the control group showing an increase of +0.98 kg/m^2^ and the intervention group a reduction of −1.25 kg/m^2^. Effect sizes were large for both parameters. ANCOVA for remaining anthropometric variables—WHR (*p* = 0.96), body fat percentage (*p* = 0.22), visceral fat (*p* = 0.84), muscle mass (*p* = 0.59), total body water (*p* = 0.22), and metabolic age (*p* = 0.61)—did not reach statistical significance after baseline adjustment, though the direction of change consistently favored the intervention group.

### 3.9. Ophthalmic Outcomes

After 6 months of follow-up, improvement in retinal condition was observed in 27.3% of patients in the dietary intervention group and 14.3% in the control group (Table 3). Stabilization/no progression was observed in 68.2% and 71.4% of patients, respectively, whereas retinal deterioration occurred in 4.5% of patients in the intervention group and 14.3% in the control group. However, the differences between groups were not statistically significant (Fisher’s exact test, *p* = 0.386).

## 4. Discussion

In the present study, we evaluated the effects of a six-month, individualized dietary intervention on body composition and retinal outcomes in patients with neovascular age-related macular degeneration (AMD) undergoing anti-VEGF therapy. The results showed a significant reduction in body weight and BMI in the intervention group, whereas a statistically significant increase in body weight was observed in the control group. No statistically significant changes were observed in the waist-to-hip ratio or total and visceral fat at the group level.

ANCOVA adjustment confirmed that the observed reductions in body weight and BMI in the intervention group were not attributable to regression to the mean or to the higher baseline body mass in this group. The divergent trajectories, weight gain in controls (+2.60 kg) versus weight loss in the intervention group (−3.09 kg), with large effect sizes (η^2^ = 0.29 and 0.24, respectively) support the conclusion that the individualized dietary intervention produced clinically meaningful improvements in body composition. The absence of significant ANCOVA effects for other body composition parameters likely reflects the modest sample size and the relatively short follow-up period rather than a true lack of effect, given that consistent directional trends were observed across all measured variables.

Nevertheless, the non-equivalent baseline characteristics between groups remain an important limitation of the study and should be considered when interpreting the observed intervention effects. Although ANCOVA adjustment was applied to reduce the influence of baseline anthropometric differences, residual confounding cannot be fully excluded due to the pilot nature of the study and the relatively small sample size.

Analysis of median values and ranges (minimum–maximum) of body weight and BMI in relation to clinical outcomes indicated that in the intervention group, patients with improved or stable retinal status exhibited reduced values of these parameters. In contrast, in the control group, despite the observed increase in body weight and unfavorable changes in some anthropometric parameters, no clear relationship was observed between these changes and clinical AMD outcomes. These findings suggest that, in the studied population, anthropometric parameters were not independent determinants of the response to anti-VEGF therapy, which may explain the divergent patterns observed in the control versus intervention groups.

These observations relate to previous studies suggesting a possible association between overweight and obesity and the risk and progression of AMD, although such a relationship was not clearly confirmed in our study [10,11,12]. Cohort studies indicate that higher BMI and waist-to-hip ratio (WHR) may correlate with an increased risk of early and late AMD, while physical activity may potentially slow disease progression [37,38]. Furthermore, excessive body weight is strongly correlated with the neovascular form of AMD [16].

In our intervention, patients followed a balanced diet rich in vegetables, fruits, whole grains, and nutrients considered beneficial for retinal health, including lutein, zeaxanthin, lycopene, antioxidant vitamins, and omega-3 fatty acids. This approach aligns with previous studies showing that diets rich in these foods may reduce the risk of progression to neovascular AMD, whereas Western or high-fructose diets may have the opposite effect [16,17,18,39]. Mediterranean or Asian-style diets rich in fruits, vegetables, legumes, and whole grains have been associated with a lower risk of neovascular AMD, while Western diets appear detrimental [18]. In animal studies, high-fructose diets induced more retinal pathological changes compared to standard diets [39]. Excessive obesity may contribute to AMD development through chronic inflammation, oxidative stress, and altered availability of lipophilic carotenoids, such as lutein and zeaxanthin, stored in adipose tissue [39,40,41,42].

Unlike AREDS-type studies, which primarily investigated dietary supplementation in earlier AMD stages, the present pilot study evaluated individualized dietary intervention in patients with neovascular AMD undergoing anti-VEGF therapy [43,44].

It should also be noted that other parameters, such as visceral fat, muscle mass, and hydration status, may play a role in AMD pathophysiology [45,46,47]. Maintenance of muscle mass may be particularly important in elderly patients, as age-related sarcopenia is associated with reduced physical function, chronic inflammation, and poorer overall health outcomes. Therefore, preservation of lean body mass may represent an additional benefit of individualized nutritional intervention in older patients with neovascular AMD [48,49]. Although no significant changes in visceral fat were observed at the group level, patients with better clinical outcomes tended to maintain or slightly increase muscle mass and total body water, highlighting the importance of overall nutritional status in older adults. Exploratory correlation analyses were also performed between selected anthropometric and biochemical parameters; however, these findings should be interpreted cautiously due to the limited sample size and pilot nature of the study.

This study has several important limitations. First, the sample size was relatively small, and nearly 50% of initially enrolled participants did not complete the study, which limited the statistical power and prevented continuation of the observation. Second, the follow-up period was limited to six months, which may be insufficient to detect meaningful changes in a chronic and slowly progressing disease such as AMD. Therefore, the absence of a statistically significant effect on retinal outcomes may reflect these methodological limitations. Further large-scale, long-term, multicenter studies are needed to more precisely assess whether improvements in body composition can meaningfully influence AMD progression.

In conclusion, the findings indicate that individualized dietary intervention can effectively improve body composition in patients with neovascular AMD, although no statistically significant impact on retinal status was observed over six months. Nevertheless, the dietary intervention group demonstrated a numerically higher frequency of retinal improvement and lower frequency of retinal deterioration compared with the control group. The results suggest that personalized nutrition may support overall health and weight management, but its direct effect on AMD progression remains inconclusive.

This prospective, controlled, multi-stage study was conducted between 2018 and 2021 at the First Department of Ophthalmology, Pomeranian Medical University, Szczecin, Poland. The study design received a positive opinion from the Bioethics Committee of the Regional Medical Chamber in Szczecin (approval no. OIL-Sz/MF/KB/452/11/06/2018). Patient participation in the study was voluntary.

## 5. Conclusions

This study demonstrates that a six-month individualized dietary intervention can effectively improve body composition in patients with neovascular age-related macular degeneration (AMD) undergoing anti-VEGF therapy, as evidenced by reductions in body weight and BMI. Despite improvements in anthropometric parameters, no statistically significant effects on retinal treatment outcomes were observed within the relatively short 6-month follow-up period. Nevertheless, the dietary intervention group demonstrated a numerically higher frequency of retinal improvement and lower frequency of retinal deterioration compared with the control group. These results suggest that, while personalized nutrition may support overall health, weight management, and potentially maintain a favorable body composition, its direct impact on AMD progression remains inconclusive. Incorporating individualized dietary counseling into routine care may be beneficial for patients’ overall health. Further long-term, large-scale studies are warranted to clarify the role of dietary interventions in modulating AMD progression.

The present study should be considered a pilot feasibility study. Potential beneficial effects of dietary modification on retinal health may require a substantially longer observation period to become clinically detectable. Changes in macular pigment density, oxidative stress, inflammatory status, and retinal metabolism are likely to occur gradually and may not be fully reflected within a six-month intervention period. Therefore, the absence of significant retinal improvement does not exclude the possibility of long-term benefits associated with individualized nutritional intervention in patients with neovascular AMD. The present pilot study may provide preliminary data for estimating sample size and designing larger long-term clinical studies evaluating the effect of dietary intervention on retinal and visual outcomes in patients with neovascular AMD.

## Figures and Tables

**Table 1:** Nutrient composition of the reference diet with an energy value of 1900 kcal.

Energy (kcal)	P (g)	P (%)	F (g)	F (%)	C (g)	C (%)	Dietary Fiber (g)	Cholesterol [mg]
1909.6	76.4	16	68	32	248	52	33	256
**Lutein + Zeaxanthin** **[µg]**	**Lycopene [µg]**	**Vitamin A [µg]**	**Vitamin E [mg]**	**Vitamin C [mg]**	**Zinc** **[mg]**	**Copper** **[mg]**	**Manganese** **[mg]**	**Selenium** **[µg]**
5582	8114	1111	9.69	248	9.02	1.68	4.5	87

Protein (P), fat (F), carbohydrates (C).

**Table 2:** Summary of statistically significant differences in anthropometric parameters, demonstrated using the Wilcoxon signed-rank test for paired groups (comparison of results before and after dietary intervention in the same patients) and the Mann–Whitney U test for independent data (comparison of data between the diet and no-diet groups, i.e., different patients).

	No Diet, Baseline $\bar{x}$ (mean ± SD)	No Diet, 6 Months $\bar{x}$ (mean ± SD)	Diet, Baseline $\bar{x}$ (mean ± SD)	Diet, 6 Months $\bar{x}$ (mean ± SD)
**Body weight (kg)**	68.59 ± 8.74 ^A,B^	71.19 ± 9.59 ^A^	81.02 ± 15.84 ^a,B^	77.93 ± 15.90 ^a^
**WHR**	0.88 ± 0.09 ^B^	0.88 ± 0.09	0.92 ± 0.08 ^B^	0.92 ± 0.08
**BMI (kg/m^2^)**	26.80 ± 2.83 ^B^	27.79 ± 2.92	30.42 ± 5.68 ^a,B^	29.17 ± 5.15 ^a^
**Body fat (%)**	36.10 ± 6.35	38.33 ± 6.51	39.98 ± 8.22	37.35 ± 8.58
**Total body water (%)**	45.90 ± 4.40	44.53 ± 4.55	44.30 ± 5.77	45.33 ± 5.82
**Muscle mass (kg)**	41.38 ± 5.43 ^B^	41.71 ± 5.46	46.67 ± 9.75 ^B^	46.12 ± 10.86
**Metabolic age**	67.24 ± 11.87	68.19 ± 12.93	73.27 ± 10.01	69.41 ± 12.11
**Visceral fat level (%)**	11.40 ± 3.42 ^B^	11.62 ± 3.47	13.70 ± 3.35 ^B^	12.86 ± 3.31

Legend: A—statistically significant differences (*p* < 0.05) in the control group before (day 0) and after 6 months of observation; a—statistically significant differences (*p* < 0.05) in the study group before (day 0) and after 6 months of dietary intervention; B—statistically significant differences (*p* < 0.05) between the study and control groups at day 0.

**Table 3:** Fisher’s exact test: *p* = 0.386.

Ophthalmic Outcome	Diet Group *n* (%)	Control Group *n* (%)
Improvement	6 (27.3%)	3 (14.3%)
No progression	15 (68.2%)	15 (71.4%)
Deterioration	1 (4.5%)	3 (14.3%)

## Data Availability

The original contributions presented in this study are included in the article. Further inquiries can be directed to the corresponding author.

## Abbreviations

The following abbreviations are used in this manuscript:

AMD	Age-related macular degeneration
BIA	Bioelectrical impedance analysis
VEGF	Vascular endothelial growth factor
AREDS	Age-related eye disease study
COVID-19	Coronavirus disease 2019
BMI	Body mass index
WHR	Waist-to-hip ratio
OCT	Optical coherence tomography
OCTA	Optical coherence tomography angiography
SOD	Superoxide dismutase
CAT	Catalase
GSH-Px	Glutathione peroxidase
MDA	Malondialdehyde

## References

[1] Jankowska-Lech I., Grabska-Liberek I., Krzyżewska-Niedziałek A., Pietruszyńska M. (2013). Zwyrodnienie Plamki Związane z Wiekiem (AMD)—Choroba Się Społeczeństw. Postępy Nauk. Med..

[2] Deng Y., Qiao L., Du M., Qu C., Wan L., Li J., Huang L. (2022). Age-Related Macular Degeneration: Epidemiology, Genetics, Pathophysiology, Diagnosis, and Targeted Therapy. Genes Dis..

[3] Kotarba A., Borowiak E., Czajkowski J. (2014). Okulistyczne Aspekty Starzenia Się Człowieka. Probl. Pielęgniarstwa.

[4] Solomon S.D., Lindsley K., Vedula S.S., Krzystolik M.G., Hawkins B.S. (2019). Anti-Vascular Endothelial Growth Factor for Neovascular Age-Related Macular Degeneration. Cochrane Database Syst. Rev..

[5] Reid C.A., Nettesheim E.R., Connor T.B., Lipinski D.M. (2018). Development of an Inducible Anti-VEGF RAAV Gene Therapy Strategy for the Treatment of Wet AMD. Sci. Rep..

[6] Rasoulinejad S.A., Maroufi F. (2021). CRISPR-Based Genome Editing as a New Therapeutic Tool in Retinal Diseases. Mol. Biotechnol..

[7] Marchesi N., Capierri M., Pascale A., Barbieri A. (2024). Different Therapeutic Approaches for Dry and Wet AMD. Int. J. Mol. Sci..

[8] Brady W.E., Mares-Perlman J.A., Bowen P., Stacewicz-Sapuntzakis M. (1996). Human Serum Carotenoid Concentrations Are Related to Physiologic and Lifestyle Factors. J. Nutr..

[9] de Koning-Backus A.P.M., Kiefte-de Jong J.C., van Rooij J.G.J., Uitterlinden A.G., Voortman T.G., Meester-Smoor M.A., Klaver C.C.W. (2023). Lifestyle Intervention Randomized Controlled Trial for Age-Related Macular Degeneration (AMD-Life): Study Design. Nutrients.

[10] Ng Yin Ling C., Lim S.C., Jonas J.B., Sabanayagam C. (2021). Obesity and Risk of Age-Related Eye Diseases: A Systematic Review of Prospective Population-Based Studies. Int. J. Obes..

[11] Peeters A. (2008). Changes in Abdominal Obesity and Age-Related Macular Degeneration. Arch. Ophthalmol..

[12] Bogdănici C.G., Bogdănici C.M., Pavel I.A., Ganea C.V., Donica V.C., Cărăușu E.M. (2025). Associations Between Nutritional Factors, Obesity and Ocular Diseases: A Narrative Literature Review. Nutrients.

[13] Mrowicka M., Mrowicki J., Kucharska E., Majsterek I. (2022). Lutein and Zeaxanthin and Their Roles in Age-Related Macular Degeneration—Neurodegenerative Disease. Nutrients.

[14] Tan B.L., Norhaizan M.E., Liew W.P.P., Rahman H.S. (2018). Antioxidant and Oxidative Stress: A Mutual Interplay in Age-Related Diseases. Front. Pharmacol..

[15] Li L.H., Lee J.C.-Y., Leung H.H., Lam W.C., Fu Z., Lo A.C.Y. (2020). Lutein Supplementation for Eye Diseases. Nutrients.

[16] Zhang Q.Y., Tie L.J., Wu S.S., Lv P.L., Huang H.W., Wang W.Q., Wang H., Ma L. (2016). Overweight, Obesity, and Risk of Age-Related Macular Degeneration. Investig. Ophthalmol. Vis. Sci..

[17] Chew E.Y., Clemons T.E., SanGiovanni J.P., Danis R., Ferris F.L., Elman M., Antoszyk A., Ruby A., Orth D., Bressler S. (2013). Lutein+ Zeaxanthin and Omega-3 Fatty Acids for Age-Related Macular Degeneration: The Age-Related Eye Disease Study 2 (AREDS2) Randomized Clinical Trial. JAMA.

[18] Age-Related Eye Disease Study Research Group (2001). A Randomized, Placebo-Controlled, Clinical Trial of High-Dose Supplementation With Vitamins C and E, Beta Carotene, and Zinc for Age-Related Macular Degeneration and Vision Loss. Arch. Ophthalmol..

[19] Carneiro Â., Andrade J.P. (2017). Nutritional and Lifestyle Interventions for Age-Related Macular Degeneration: A Review. Oxidative Med. Cell. Longev..

[20] Ulańczyk Z., Grabowicz A., Cecerska-Heryć E., Śleboda-Taront D., Krytkowska E., Mozolewska-Piotrowska K., Safranow K., Kawa M.P., Dołęgowska B., Machalińska A. (2020). Dietary and Lifestyle Factors Modulate the Activity of the Endogenous Antioxidant System in Patients with Age-Related Macular Degeneration: Correlations with Disease Severity. Antioxidants.

[21] Chapman N.A., Jacobs R.J., Braakhuis A.J. (2019). Role of Diet and Food Intake in Age-Related Macular Degeneration: A Systematic Review. Clin. Exp. Ophthalmol..

[22] Kaur D., Rasane P., Singh J., Kaur S., Kumar V., Mahato D.K., Dey A., Dhawan K., Kumar S. (2019). Nutritional Interventions for Elderly and Considerations for the Development of Geriatric Foods. Curr. Aging Sci..

[23] Fairfield K.M., Fletcher R.H. (2002). Vitamins for Chronic Disease Prevention in Adults. JAMA.

[24] Eisenhauer B., Natoli S., Liew G., Flood V. (2017). Lutein and Zeaxanthin—Food Sources, Bioavailability and Dietary Variety in Age-Related Macular Degeneration Protection. Nutrients.

[25] Johnson E.J., Maras J.E., Rasmussen H.M., Tucker K.L. (2010). Intake of Lutein and Zeaxanthin Differ with Age, Sex, and Ethnicity. J. Am. Diet. Assoc..

[26] Jarosz M. (2012). Normy Żywienia dla Populacji Polskiej-Nowelizacja.

[27] Rao A.V., Shen H. (2002). Effect of Low Dose Lycopene Intake on Lycopene Bioavailability and Oxidative Stress. Nutr. Res..

[28] Institute of Medicine (US) Panel on Micronutrients (2001). Dietary Reference Intakes for Vitamin A, Vitamin K, Arsenic, Boron, Chromium, Copper, Iodine, Iron, Manganese, Molybdenum, Nickel, Silicon, Vanadium, and Zinc.

[29] Costa-Camilo E., Cardoso F., Duarte I., Carvalho G.P., de Almeida J.M.G.C.F., Sobral R.G., Pinheiro C. (2025). Mediterranean Diet as a Nutraceutical and Sustainable Model for Health and Environmental Wellbeing. Gastronomy.

[30] Ristori S., Bertoni G., Bientinesi E., Monti D. (2025). The Role of Nutraceuticals and Functional Foods in Mitigating Cellular Senescence and Its Related Aspects: A Key Strategy for Delaying or Preventing Aging and Neurodegenerative Disorders. Nutrients.

[31] Akram M., Munir N., Daniyal M., Chukwuebuka E., Găman M., Onyekere P.F., Olatunde A., Egbuna C., Dable-Tupas G. (2020). Functional Foods and Nutraceuticals. Bioactive Components, Formulations and Innovations.

[32] Wu Y., Xie Y., Yuan Y., Xiong R., Hu Y., Ning K., Ha J., Wang W., Han X., He M. (2023). The Mediterranean Diet and Age-Related Eye Diseases: A Systematic Review. Nutrients.

[33] Cohen J. (2013). Statistical Power Analysis for the Behavioral Sciences.

[34] Rychlik E., Stoś K., Woźniak A., Mojskiej H. (2024). Normy Żywienia dla Populacji Polski.

[35] (2011). Waist Circumference and Waist-Hip Ratio: Report of a WHO Expert Consultation, Geneva, 8–11 December 2008.

[36] TANITA Corporation (2015). Body Composition Guide for Inner Scan.

[37] Seddon J.M., Cote J., Davis N., Rosner B. (2003). Progression of Age-Related Macular Degeneration Association With Body Mass Index, Waist Circumference, and Waist-Hip Ratio. Arch. Ophthalmol..

[38] Adams M.K.M., Simpson J.A., Aung K.Z., Makeyeva G.A., Giles G.G., English D.R., Hopper J., Guymer R.H., Baird P.N., Robman L.D. (2011). Abdominal Obesity and Age-Related Macular Degeneration. Am. J. Epidemiol..

[39] Thierry M., Pasquis B., Acar N., Grégoire S., Febvret V., Buteau B.N., Gambert-Nicot S., Bron A.M., Creuzot-Garcher C.P., Bretillon L. (2014). Metabolic Syndrome Triggered by High-Fructose Diet Favors Choroidal Neovascularization and Impairs Retinal Light Sensitivity in the Rat. PLoS ONE.

[40] Ponti F., Santoro A., Mercatelli D., Gasperini C., Conte M., Martucci M., Sangiorgi L., Franceschi C., Bazzocchi A. (2020). Aging and Imaging Assessment of Body Composition: From Fat to Facts. Front. Endocrinol..

[41] Rea I.M., Gibson D.S., McGilligan V., McNerlan S.E., Denis Alexander H., Ross O.A. (2018). Age and Age-Related Diseases: Role of Inflammation Triggers and Cytokines. Front. Immunol..

[42] Fontana L., Eagon J.C., Trujillo M.E., Scherer P.E., Klein S. (2007). Visceral Fat Adipokine Secretion Is Associated With Systemic Inflammation in Obese Humans. Diabetes.

[43] Clemons T.E., Milton R.C., Klein R., Seddon J.M., Ferris F.L. (2005). Risk Factors for the Incidence of Advanced Age-Related Macular Degeneration in the Age-Related Eye Disease Study (AREDS)AREDS Report No. 19. Ophthalmology.

[44] Camelo S., Latil M., Veillet S., Dilda P.J., Lafont R. (2020). Beyond AREDS Formulations, What Is Next for Intermediate Age-Related Macular Degeneration (IAMD) Treatment? Potential Benefits of Antioxidant and Anti-Inflammatory Apocarotenoids as Neuroprotectors. Oxidative Med. Cell. Longev..

[45] Haas P., Kubista K.E., Krugluger W., Huber J., Binder S. (2015). Impact of Visceral Fat and Pro-inflammatory Factors on the Pathogenesis of Age-related Macular Degeneration. Acta Ophthalmol..

[46] Choi K.-E., Joung C., Pahk K.J., Kim H., Pahk K. (2024). Metabolic Activity of Visceral Adipose Tissue Is Associated with Age-Related Macular Degeneration: A Pilot 18F-FDG PET/CT Study. Front. Endocrinol..

[47] Edmonds C.J., Foglia E., Booth P., Fu C.H.Y., Gardner M. (2021). Dehydration in Older People: A Systematic Review of the Effects of Dehydration on Health Outcomes, Healthcare Costs and Cognitive Performance. Arch. Gerontol. Geriatr..

[48] Katipoğlu Z., Katipoğlu B., Turan M. (2025). Association of Sarcopenia with Age-Related Macular Degeneration in the Very Elderly. Cukurova Med. J..

[49] Wang S., Hong Y., Qu Y., Zheng K., Luo H., Chen R., Jia H., Liu X., Sun X. (2025). Association between Low Handgrip Strength and the Increased Risk of Age-Related Macular Degeneration: Results from UK Biobank Cohort Study. Aging Clin. Exp. Res..

